# Evolution of asexual *Daphnia pulex* in Japan: variations and covariations of the digestive, morphological and life history traits

**DOI:** 10.1186/s12862-019-1453-9

**Published:** 2019-06-13

**Authors:** Xiaofei Tian, Hajime Ohtsuki, Jotaro Urabe

**Affiliations:** Graduate School of Life Sciences, Tohoku University, 6-3 Aramaki aza Aoba, Sendai, 980-8578 Japan

**Keywords:** *Daphnia*, Digestive traits, Genetic distance, Heritability, Invasive species, Life history traits, Morphological traits, Phenotypic distance

## Abstract

**Background:**

Several genetic lineages of obligate parthenogenetic *Daphnia pulex*, a common zooplankton species, have invaded Japan from North America. Among these, a lineage named JPN1 is thought to have started colonization as a single genotype several hundred to thousand years ago and subsequently produced many genotypes in Japan. To examine the phenotypic variations due to ecological drivers diverging the genotypes in new habitats, we measured heritability and variation in 17 traits, including life history, morphology and digestive traits, and the genetic distance among the *D. pulex* JPN1 genotypes in Japan.

**Results:**

We found that most of the traits measured varied significantly among the genotypes and that heritability was highest in the morphological traits, followed by the digestive and life history traits. In addition, 93% of the variation in these traits was explained by the first three components in the principal component analysis, implying that variation of these heritable traits is not random but rather converged into a few directions. These relations among traits revealed the potential importance of predation pressures and food conditions as factors for diverging and selecting different genotypes. However, the magnitude of the difference in any single trait group did not correlate with the genetic distance.

**Conclusions:**

Our findings show that the divergent traits evolved within *D. pulex* JPN1 lineage without genetic recombination, since their ancestral clone invaded Japan. Large variations and covariations of the phenotypic traits, irrespective of the genetic distance among the genotypes, support the view that the invasive success of *D. pulex* JPN1 was promoted by a genetic architecture that allowed for large phenotypic variations with a limited number of functionally important mutations without recombination.

**Electronic supplementary material:**

The online version of this article (10.1186/s12862-019-1453-9) contains supplementary material, which is available to authorized users.

## Background

Variations and relationships among heritable traits provide clues for identifying selective forces and thus understanding the evolution of species [1–3]. Specifically, if heritable traits diverged among isolated populations that shared common ancestral individuals, then we can analyse how phenotypic variation is related to genetic variation in terms of the ecological drivers promoting evolution. In general, populations of invasive species are founded by a few common ancestral individuals. Thus, examination of the phenotypic and genetic variations among these invasive populations is useful for unveiling the evolutionary processes that take place during the adaption to new habitats [4, 5].

Several genetic lineages of panarctic *Daphnia pulex* [6], a common zooplankton species, have invaded Japan from North America. Among these, a lineage named JPN1 is thought to have started the colonization as a single genotype several hundred to thousand years ago [7]. Although individuals of *D. pulex* JPN1 are obligate parthenogenetic animals, several genotypes (clones) were found within this lineage, suggesting that they genetically evolved without recombination after the initial invasion and likely adapted to new habitats in Japan [7]. Since they reproduce asexually, their populations are established by and composed of single genotypes [8, 9]. If phenotypic traits vary among these genotypes (clones) and are heritable, then the genotypes of *D. pulex* that invaded Japan are a good model to explore whether the magnitude of the difference in the phenotypic traits is related to genetic distance among the genotypes and what environmental factors promoted evolution after *Daphnia* invasions into new habitats.

In nature, the abundance of *Daphnia* individuals is greatly influenced by a variety of environmental and biological factors, including interspecies interactions [10–12]. Among these, predation is one of the important factors affecting *Daphnia* populations since it directly affects the survival rate [13–16] and indirectly affects reproduction when the survivors mature [17, 18]. In addition, *Daphnia* populations frequently suffer from deficiencies in the quantity and quality of algal food [19–21]. Therefore, several studies have examined intraspecific or genotype-specific differences in morphological and life history traits of *Daphnia* species under different predation pressures [22–24] and food conditions [1, 25–28]. These studies showed that although the magnitude of variation caused by predators and food conditions differed among the phenotypic traits, the response of most traits was, to some extent, genotype specific. This suggests that at least some life history and morphological traits are heritable and that predation and food condition may have played crucial roles in selecting genotypes with different phenotypic traits, such as growth rate and maturation size.

To maximize their fitness under given food scarcity conditions with or without the presence of predators, animals must efficiently gain energy and nutrients from their food. Recent studies have suggested that in addition to a herbivore’s feeding rate and digestive capacity, digestive enzyme activity is important for the animal’s ability to cope with nutritionally suboptimal foods and maximize its fitness [29, 30]. Thus, under different food conditions, genotypes with different digestive capacities are likely selected. Previous studies have shown that digestive enzyme activities are highly associated with the expression of related genes [31, 32]. However, few studies have examined whether digestive enzyme activity differs among genotypes and is thus a heritable trait.

In obligate parthenogenetic organisms, such as asexual individuals of *D. pulex*, the variation and relationship among phenotypic traits have noticeable implications for their evolution [33]. The substantial linkage of genes in asexual populations makes it impossible for a phenotypic trait to evolve independently of other traits [34, 35]. Accordingly, genotypes that are successfully maintained by given environmental conditions have likely both adaptive and non-adaptive traits [36, 37]. If this is the case, then these traits are likely linked to each other across asexual genotypes. Conversely, if heritable differences in traits diverged evolutionally, without pleiotropic and epistatic effects [38], then it is likely that with an increasing number of base substitutions (mutations), the phenotypes will become more dissimilar among the genotypes. However, little is known about if and how life history, morphological and digestive traits are covaried in *Daphnia* [1] and how phenotypic differences in these traits are related to the genetic distance among genotypes.

To address these uncertainties, we examined the heritabilities of life history, morphology and digestive traits of *D. pulex* JPN1 genotypes. The heritability is a metrics measuring the degree of genetic attribute to a phenotypic resemblance between parental and offspring individuals in sexual organisms [2]. In the same sense, we used this term as the degree of genetic attribute to phenotypic similarity of a trait among asexual genotypes sharing the same ancestor individual. Our specific objectives were to clarify (1) if not only life history and morphology traits but also digestive traits are heritable, (2) how much these traits vary in response to changes in food conditions, (3) how much these traits covary with each other, and (4) if the magnitude of the phenotypic difference in heritable traits relates to individual genetic distance. By examining these variables, we explore the evolutionary ability of the phenotypic traits and ecological drivers that cause divergence in the phenotypes of a *D. pulex* lineage after invading Japan.

## Results

### Effects of genotype and food level on phenotypic traits

Both genotype (G) and food level (F) significantly affected 11 out of 17 traits (Table 1). Conversely, the growth coefficient *k* and the body length at maturation and at day 5 only differed among genotypes, the intermoult duration before maturation differed between food levels, and the lipase activity and intermoult duration after maturation did not differ among genotypes or food levels (Table 1). Compared with these main effects, significant interaction effects of genotype and food level (GxF) were found for a limited number of traits including beta-glucosidase and phosphatase activities for digestive traits and neonate size for morphological traits. Variances explained by genotype and food level differed highly among the traits. For example, > 60% of the variation in day 5 body length and relative tail-spine length were explained by the genotype, while the large variations in beta-glucosidase, arginine amino-peptidase, mean egg number, maturation age and maximum body length were explained by the present food level (Table 1). Thus, the effects of genotype and food level varied among the traits, even within the same trait category. However, on average, the proportion of the variance that was explained by the genotype was greater in morphological traits (mean 39.50%) than in either digestive traits (mean 6.28%) or life history traits (mean 10.66%) (Table 1).

**Table 1:** The effects of genotype, food level, and their interaction on phenotypic traits in *Daphnia pulex*

Trait	Mean	Range	Genotype			Food			Genotype × Food		
			F-value	*p*	*Var %*	F-value	*p*	*Var %*	F-value	*p*	*Var %*
Digestive											
Beta-glucosidase (nmol hr.^−1^ mg_protein_^−1^)	322.8	93.9–680.6	10.56	**< 0.001**	4.42	260.46	**< 0.001**	83.19	4.32	**0.03**	1.77
Lipase (nmol hr.^−1^ mg_protein_^− 1^)	1949.4	1212.0–2789.4	1.61	0.95	0.00	0.02	1.00	0.00	0.99	1.00	0.00
Alkaline phosphatase (nmol hr.^−1^ mg_protein_^− 1^)	764.47	325.5–1526.2	13.83	**< 0.001**	2.08	37.56	**< 0.001**	35.41	6.15	**0.01**	20.83
Arginine amino-peptidase (nmol hr.^−1^ mg_protein_^− 1^)	195.93	67.0–578.5	6.46	**0.01**	8.80	125.17	**< 0.001**	80.00	0.38	1.00	0.00
Alanine amino-peptidase (nmol hr.^−1^ mg_protein_^− 1^)	178.05	82.9–310.1	11.75	**< 0.001**	16.12	106.94	**< 0.001**	59.16	0.68	1.00	0.40
Life history											
Maturation age (days)	7.62	5.00–12.00	9.48	**< 0.001**	12.55	96.81	**< 0.001**	65.21	0.20	1.00	0.00
Maturation instar number	3.14	1.00–6.00	4.66	**0.02**	10.40	36.65	**< 0.001**	45.60	0.96	1.00	0.09
Maturation body length (mm)	1.61	1.32–1.90	6.45	**< 0.001**	23.57	7.52	0.09	14.35	0.74	1.00	0.00
Intermoult duration before maturation (days)	1.94	1.50–4.50	1.62	1.00	4.21	9.56	**0.03**	20.20	0.05	1.00	0.00
Intermoult duration after maturation (days)	2.31	2.00–2.67	1.65	1.00	0.00	3.10	0.88	4.98	2.61	0.44	11.20
*k*	0.11	0.06–0.17	5.57	**0.009**	19.40	3.60	0.81	0.05	1.61	1.00	0.05
Mean egg number of the first three clutches	6.19	1.67–12.50	7.78	**< 0.001**	4.51	177.12	**< 0.001**	76.21	2.78	0.33	3.99
Morphology											
Mean neonate size of the first three clutches (mm)	0.63	0.50–0.78	10.28	**< 0.001**	1.47	19.59	**< 0.001**	13.64	8.43	**< 0.001**	29.32
Body length at five days (mm)	1.29	0.98–1.54	21.26	**< 0.001**	64.66	2.16	0.30	1.37	1.18	0.67	1.64
Body weight at five days (mg)	0.02	0.006–0.03	15.52	**< 0.001**	47.37	11.84	**0.001**	15.79	1.72	0.34	5.26
Relative tail spine length of the first adult instar	0.19	0.10–0.25	41.78	**< 0.001**	75.67	18.87	**< 0.001**	0.00	2.49	0.66	5.88
*L*_*∞*_ (mm)	2.45	1.89–3.10	12.09	**< 0.001**	8.33	158.07	**< 0.001**	70.43	3.35	0.22	5.24

The means and ranges of these traits examined for *Daphnia* JPN1 are shown with the variance components of each effect (*Var* %). Statistically significant *p*-values (< 0.05) are shown in bold

### Heritability and genetic variance

Broad-sense heritability varied highly among the traits and ranged from 0 to 0.91 with a mean of 0.33 (Table 2). Among the three trait categories, heritability greater than 0.5 was often found in morphological traits but not in life history traits. Digestive traits showed relatively high heritability, except for lipase activity. The coefficients of genetic variation for the digestive traits were comparable to those of the morphological traits and were generally higher than those of the life history traits. In life history traits, maturation age showed the highest heritability, followed by maturation size and the growth coefficient (k). Since heritability was less than 0.1 for the intermoult durations of mature and immature individuals, and lipase activity showed no significant difference among the genotypes, we excluded these traits from the following analyses.

**Table 2:** Genetic variance, broad-sense heritability, 95% confidence interval of heritability and coefficient of variation for 17 *Daphnia* phenotypic traits

Trait	Genetic variance (V_g_)	Broad-sense heritability (H^2^)	95% confidence interval of heritability (CI)	Coefficient of variation (CV) (%)
Digestive				
Beta-glucosidase	1630.00	0.35	(0.00, 0.31)	12.51
Lipase	8489.419	0.07	(0.00, 0.17)	38.42
Alkaline phosphatase	13,060.00	0.36	(0.00, 0.63)	14.95
Arginine amino-peptidase	2128.45	0.41	(0.28, 0.80)	23.55
Alanine amino-peptidase	845.20	0.56	(0.48, 0.91)	16.33
Life history				
Maturation age	0.48	0.41	(0.10, 0.73)	9.08
Maturation instar number	0.13	0.20	(0.01, 0.61)	11.64
Maturation body length	0.004	0.28	(0.00, 0.08)	3.92
Intermoult duration before maturation	0.001	0.09	(0.00, 0.48)	1.95
Intermoult duration after maturation	0.00	0.00	(0.00, 0.19)	0.00
*k*	0.0002	0.12	(0.04, 0.71)	12.86
Mean egg number of the first three clutches	0.28	0.23	(0.00, 0.57)	8.54
Morphology				
Mean neonate size of the first three clutches	0.00006	0.04	(0.00, 0.08)	1.22
Body length at five days	0.02	0.67	(0.66, 0.85)	9.83
Body weight at five days	0.00002	0.58	(0.66, 0.87)	27.61
Relative tail spine length of the first adult instar	0.001	0.91	(0.44, 0.72)	17.99
*L*_*∞*_	0.01	0.31	(0.00, 0.57)	4.41

### Relationship among phenotypic traits

**Fig. 1:**
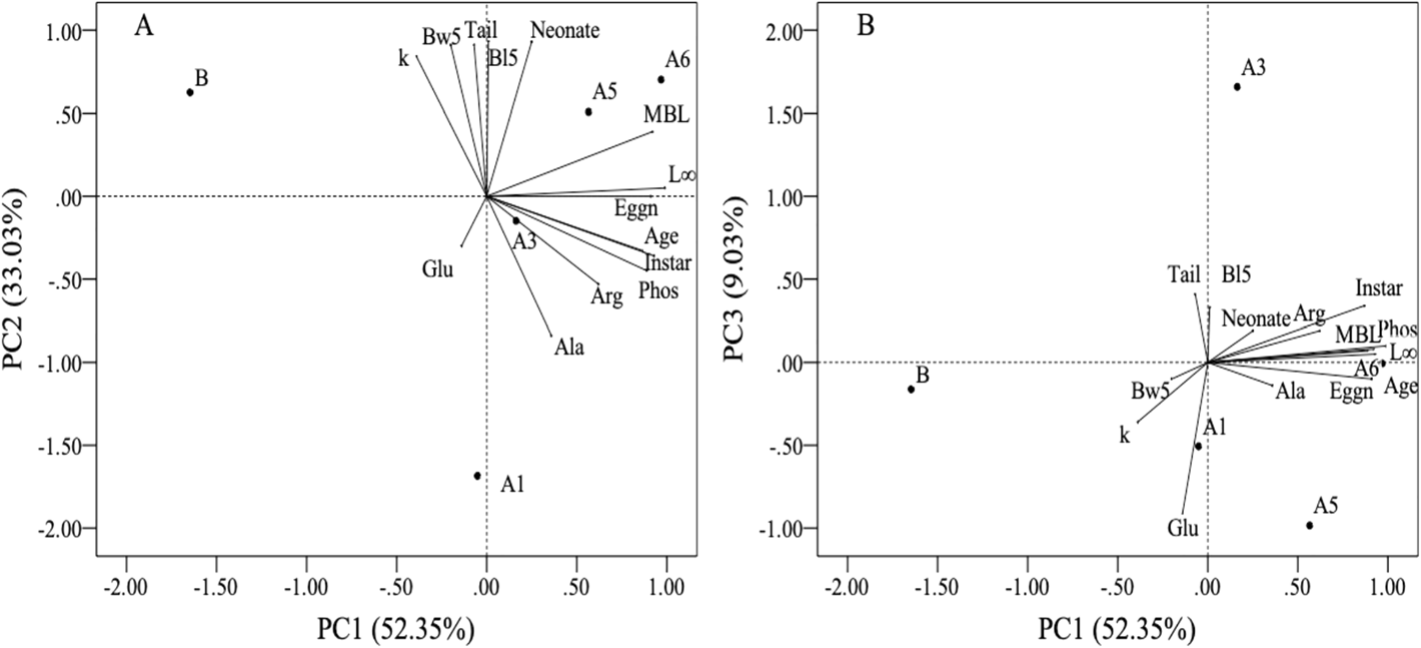
Results of PCA showing a biplot of PC scores of JPN1 genotypes (A1, A3, A5, A6 and B) and loadings of phenotypic traits (Glu: beta-glucosidase; Phos: alkaline phosphatase; Arg: arginine amino-peptidase; Ala: alanine amino-peptidase; Age: maturation age; Instar: maturation instar number; MBL: maturation body length; k: growth coefficient; Eggn: mean egg number of the first three clutches; Neonate: mean neonate size of the first three clutches; Bl5: body length at five days; Bw5: body weight at five days; Tail: relative tail-spine length of the first adult instar; L_∞_: asymptotic body length). Panels **a** and **b** represent biplots of the PC1 and PC2, and the PC1 and PC3, respectively

Relationships among the traits were examined by principal component analysis (PCA) using best linear unbiased predictors (BLUPS). The first three components explained 93% of the variation in the traits examined. The eigenvectors of traits for each PCA showed that most of the life history traits contributed to the PC1, while most of the morphological traits contributed to the PC2. Digestive traits were not clustered together but distributed into different PCs (Table 3): alkaline phosphatase and arginine amino-peptidase activities were strongly and moderately related to PC1, while alanine amino-peptidase and beta-glucosidase activities were negatively related to PC2 and PC3, respectively. The scores of these PCs were largely different among genotypes, indicating that a suite of these phenotypic traits differed among the genotypes (Fig. 1).

**Table 3:** Factor loading, eigenvalue and variance explained by the BLUPs of heritable phenotypic traits in the principal component analysis. Components with eigenvalues greater than 1.0 have been extracted to explain the variability of phenotypic traits

Phenotypic traits	Component		
	1	2	3
*L*_*∞*_	**0.99**	0.05	0.10
Maturation age	**0.93**	−0.36	0.05
Maturation body length	**0.92**	0.39	0.08
Mean egg number of the first three clutches	**0.91**	0.00	−0.10
Alkaline phosphatase	**0.89**	−0.45	0.07
Maturation instar number	**0.87**	−0.33	0.34
Arginine amino-peptidase	0.62	−0.53	0.19
Body length at five days	0.01	**0.93**	0.33
Mean neonate size of the first three clutches	0.25	**0.93**	0.19
Body weight at five days	−0.20	**0.91**	−0.10
Relative tail spine length of the first adult instar	−0.07	**0.91**	0.41
Alanine amino-peptidase	0.36	**−0.84**	−0.14
*k*	−0.39	**0.84**	−0.36
Beta-glucosidase	−0.14	−0.30	**− 0.91**
Eigenvalue	7.33	4.62	1.12
Variance contribution rate (%)	52.35	33.03	8.03
Cumulative contribution rate (%)	52.35	85.38	93.41

The loading scores greater than 0.80 and the highest compared to each of the other components are shown in bold

### Relationship between phenotypic similarity and individual genetic distance

**Fig. 2:**
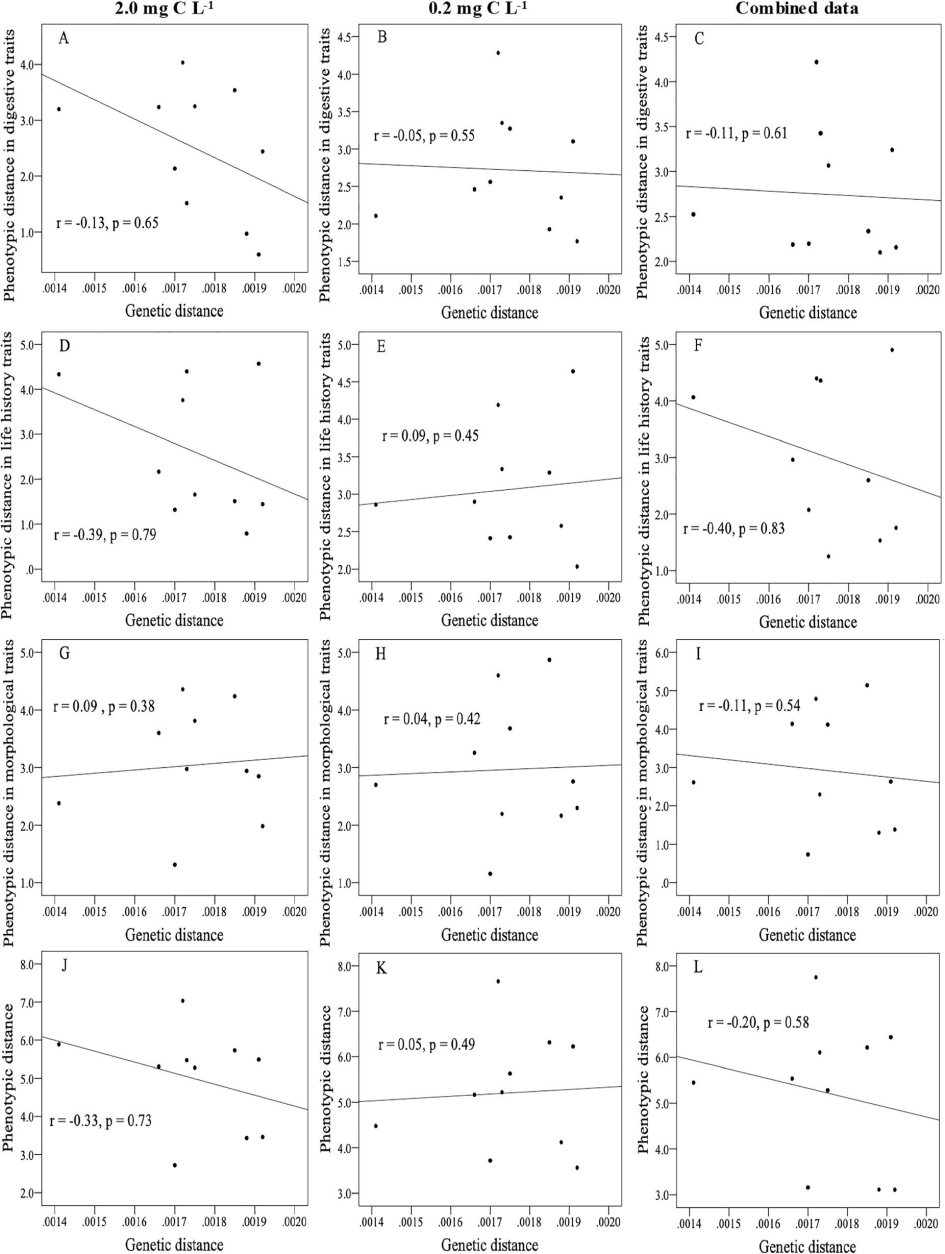
Relationship between genetic distance and phenotypic distance in different trait types between *Daphnia pulex* clones. Among these, **a**, **d**, **g**, and **j** showed the relations at the food concentrations of 2.0 mg C L^− 1^, **b**, **e**, **h**, and **k** showed the relations at the food concentrations of 0.2 mg C L^− 1^, and **c**, **f**, **i**, and **l** showed the relations in the combined data. The correlation coefficient (r) and probability of significance (p) in the Mantel test are shown in each panel

We estimated pairwise genetic distance among the genotypes according to their phylogenetic relationships (Additional file 1: Figure S1 and Additional file 2: Table S1). Then, we examined the relationships between the genetic distance and phenotypic differences in the overall traits, the trait groups (Additional file 3: Table S2) and single traits (Additional file 4: Table S3). No significant relationships were found between the genetic distance and any type of phenotypic trait examined (Fig. 2 and Additional file 5: Table S4).

## Discussion

This study showed large variations in the expression of phenotypic traits among five asexual genotypes belonging to the JPN1 clade of *D. pulex* (as defined in So et al. [7]). Most of these phenotypic traits were significantly different among the genotypes and were heritable. So et al. suggested that these genotypes share the ancestral clone that originally invaded Japan [7]. In addition, So et al. [7] used a known mutation rate of mitochondrial deoxyribonucleic acid (mtDNA) [39] and number of base substitutions among JPN1 genotypes to estimate that an ancestral clone of the JPN1 clade arrived in Japan 680~2280 years ago [7]. Although this estimation has large uncertainty, it is not divergent from the phylogenetic relationship constructed from the data of 5282 single nucleotide polymorphisms (SNPs) in this study; therefore, these genotypes certainly share the same ancestral genotype. The possibility cannot be ruled out that different genotypes invaded Japan from a remote habitat by a single event or vector. However, if this were the case, then these genotypes were phenotypically similar to each other since they were produced from obligate parthenogenesis and likely invaded from a native habitat with a unique set of selection forces. Thus, considering the large variation in the heritable phenotypic traits, it is likely that the JPN1 genotypes ecologically diverged with various phenotypic traits since they invaded Japan.

### Heritability of *Daphnia* traits

According to Mousseau & Rolf [40], who examined data from 75 species, the heritability of physiological traits is the same as that of morphological traits, which are both higher than that of life history traits. In general, it is expected that the responses of morphological and physiological traits to selection are faster than those of life history traits since the number of genes related to phenotypic variations are likely smaller in the former [41]. Since digestive enzyme activity is a physiological trait, it seems to be regulated by fewer genes compared with life history traits. Indeed, recent studies have shown that some digestive enzyme activities are highly related to the expression of a limited number of genes [31, 32]. Nonetheless, contrary to expectations, the heritability of the enzyme activities examined was lower than that of the morphological traits, although some of these showed slightly higher heritability than the life history traits. In this study, we estimated broad-sense heritability in asexual *D. pulex* genotypes since narrow-sense heritability is meaningless for asexual organisms [2]. Thus, caution is needed in understanding the implications of the heritability examined [42]. The low heritability determined in this study means that the variation is relatively lower among genotypes than among individuals within the genotypes. If all the genotypes have evolved under the same food environmental conditions, the digestive enzyme activities should have converged to the same levels among the genotypes. In addition, if food conditions vary temporally, it is disadvantageous for animals to evolve specific digestive traits. In nature, the abundance and species composition of algae that *Daphnia* prey on change not only seasonally but also spatially depending on trophic conditions [43]. Accordingly, although most of the enzyme activities were significantly different among genotypes, their heritability and thus their variation among the genotypes would be limited. Note that this differs somewhat from morphological and life history traits; digestive traits were related to different PCs depending on the following enzymes: lipase, alkaline phosphatase and arginine amino-peptidase activities were related to PC1, while alanine amino-peptidase and beta-glucosidase were related to PC2 and PC3, respectively. These results suggest that JPN1 genotypes have evolved under variable food conditions that impeded genetic linkage among digestive traits.

### Genetic relations of traits and selective forces

In this study, 93% of the variation in phenotypic traits among the JPN1 clones was summarized by the first three PCs in the principal component analysis, suggesting that the direction of these variations is not random but converged into a few directions. The phenotypic traits, maturation age, instar and size, egg numbers for the first several clutches, and asymptotic size (*L*_*∞*_) were positively correlated with each other. These results imply that clones that matured at an earlier age were of smaller size and produced a smaller number of eggs per clutch. These relationships suggest that maturation instar and size, egg number in the first several clutches and asymptotic size are determined solely by age when the *Daphnia* JPN1 clones mature. Thus, differences in these traits among clones may be a result of pleiotropic effects of mutation(s) that occurred in maturation-related genes, such as hormone genes and other endocrine genes [44], rather than the additive effects of multiple genes related to maturation.

Among the heritable digestive enzyme activities, alkaline phosphatase was positively related to maturation and clutch sizes. A similar result was obtained for arginine amino-peptidase activity, but its relation with PC1 was not as strong. It should be noted that alkaline phosphatase was not limited to digestive enzymes [45]. However, since phosphorus is a key element for promoting body mass synthesis [46], genotypes with greater alkaline phosphatase activity may have been able to mature at larger sizes and produce larger numbers of eggs. This possibility implies that genes that regulate digestive enzyme activities are functionally linked with those regulating maturation.

Studies have shown that maturation size is a crucial factor in determining the colonization success of *Daphnia* in given habitats [18, 47, 48]. In aquatic ecosystems, most zooplanktivorous fish prey preferentially on large zooplankton individuals [49, 50]. Therefore, in habitats inhabited by fish, individuals with early maturation have a higher probability of producing offspring [14, 50, 51]. In addition, it may be advantageous for *Daphnia* individuals not to grow continually and thus to have a small asymptotic size (*L*_*∞*_) for maximizing their fitness in habitats with planktivorous fish. Heritable differences in maturation age and size suggest that habitat-specific variations in predation pressures may have acted as a selective force for the diverging JPN1 genotypes.

Apart from life history traits, most morphological traits such as body size (length and weight) at day 5, neonate size, tail-spine length and growth coefficient (k) were positively related to the 2nd PC. These results imply that genotypes with higher growth rates produce larger neonates with longer tail-spines. In small lakes and ponds where planktivorous fish were not yet abundant or were absent due to temporal drying or summer or winter kills [52], invertebrate predators such as midge larvae often dominated [53, 54]. Since these invertebrate predators prey on small zooplankton, *Daphnia* individuals who rapidly attain larger sizes by postponing maturation have an advantage in reducing predation risk [17, 50, 55]. In addition, a longer tail-spine is known to be effective for *Daphnia* individuals to defend against predation by invertebrate predators [10, 56]. Thus, being larger at birth and developing longer tail-spines seem to be a defensive strategy against invertebrate predation. As above, we argue that early maturation is a life history strategy for reducing size-specific mortality imposed by fish predation. However, these life history traits are not correlated with those of morphological traits in JPN1 genotypes. This fact suggests that traits for reducing predation risks from planktivorous fish and from invertebrate predators may have evolved separately among the *D. pulex* JPN1 genotypes.

Among the enzyme activities measured, the alanine amino-peptidase activity was negatively related to PC2, indicating that genotypes with higher growth rates had reduced activity of the enzyme. Thus, the difference in the activity of this enzyme among genotypes may be a result of a decrease in activity associated with increasing body size. Nonetheless, this covariation is somewhat puzzling since animals with higher growth rates should require more nitrogen [57, 58]. One possibility is that in nature, *D. pulex* JPN1 genotypes may have experienced protein deficiencies in different manners. Although the cultured algae used in this study were sufficient in terms of nitrogen content relative to the demand of *Daphnia* [30], algal elemental and organic matter contents vary highly in natural lakes [20, 59, 60]. Thus, JPN1 genotypes with high digestive enzyme activity for amino acids may have been selected under food conditions that were deficient in protein. Alternatively, but not exclusively, in nature, *Daphnia* often experience deficiencies in biologically important chemicals such as phosphorus and essential fatty acids [20, 30]. Under such conditions, it is disadvantageous for individuals to have high nitrogen digestion abilities since they have to then dispose of excess, assimilated chemicals to maintain their body stoichiometry [61–64]. This possibility implies that JPN 1 genotypes with low digestive enzyme activities for amino acids may have been selected under food conditions that were rich in proteins to increase their growth rates and tail-spine lengths to overcome invertebrate predation risks.

In this study, only beta-glucosidase activity was related to PC3. Since we could not rule out the possibility that this trait was directly or indirectly related to other traits that were not examined in this study, it was difficult to identify selective agents causing the genetic variation of this trait. However, the results showed that variation in this trait had evolved irrespective of the selective pressures posed by predators.

### Phenotypic divergence and genetic distance

Although a number of studies have examined how phenotypic differentiation is related to genetic differentiation in populations [1, 65, 66], few have examined how phenotypic dissimilarities are related to genomic differences among individuals. If variations of heritable phenotypes occurred mainly due to mutations in additive genes or polygenes, then it is expected that increasing heritable genomic differences will result in increasingly dissimilar heritable phenotypes. Relatedly, Burstin & Charcosset [67] argued that relationships between genetic distances gauged by neutral genetic markers and phenotypic differentiation should follow a triangular pattern since large phenotypic variations are associated with large genetic variations, while small phenotypic variations are not always associated with small degrees of genetic variation. However, if large phenotypic variations emerge mainly as a result of pleiotropic effects of mutations in regulatory gene(s), then phenotypic differences would not relate to genomic distance. Supporting the latter case, neither the pairwise dissimilarities of any phenotypic categories nor the pairwise differences of any single phenotypic traits were significantly related to the pairwise genetic distances at the whole genome level (Fig. 2 and Additional file 5: Table S4). Plots of the phenotypic differences and dissimilarity against the genetic distance did not show the triangular pattern. Although our data are limited, the results suggest that large phenotypic variants of JPN1 clones have evolved mainly due to the pleiotropic effects of a limited number of mutations rather than a gradual accumulation of mutations in additive genes and polygenes.

## Conclusions

This study showed that *D. pulex* JPN1 evolved divergent traits without genetic recombination for several hundred to thousand years since their ancestral clone originally invaded Japan. Variations in heritable traits suggest that predation pressures and food environments have played roles in the divergence and selection of these clones. Moreover, the relationship among these traits support the view that large phenotypic differences among JPN1 genotypes likely occurred mainly by pleiotropic effects of a limited number of mutated genes, rather than an “adaptive character complex” that was created by natural selection favouring certain combinations of genetically independent traits through independent mutations and recombination [68]. Similar to the present results, a lineage of *Daphnia pulex* and a green alga *Caulerpa racemosa* have successfully invaded and expanded their distributions in Africa [69] and in the Mediterranean [70], respectively, although they also do not reproduce sexually. The success of asexual organisms in new habitats is often attributable to their plasticity in phenotypic traits [71]. However, regardless of the magnitude of the plasticity, asexual individuals have limited adaptive capacity if their niche is frozen [72, 73]. Other than such phenotypic plasticity, Lee [4] suggested that genetic architecture within a genotype promoting high degrees of evolvability is a prerequisite for successful colonization by invasive species. This study supports such a view: a genetic architecture allowing for variations and covariations in heritable phenotypic traits present in *D. pulex* JPN1, which was established before invading Japan, may have produced various genotypes in the lineage that could adapt rapidly to a variety of novel lakes and ponds in Japan without genetic recombination by sexual reproduction. It is necessary to identify mutated genes among JPN1 clones to uncover the genetic architectures and linkages that promote such phenotypic divergence.

## Methods

### Experimental materials

Four distinct genetic groups (JPN1–4) of obligate parthenogenetic *D. pulex* are distributed in Japan [7]. Among these, JPN1 is estimated to have invaded Japan several hundred to thousand years ago and consists of several haplotypes [7]. In this study, we used five genotypes of the *Daphnia pulex* JPN1 lineage that were previously collected from ponds and small lakes in Japan and examined in So et al. [7]. Genotype A1 was collected from Lake Hataya Ohnuma (Yamagata Prefecture, latitude (N) 38.245° longitude (E) 140.204°), A3 from Osawa Tame-ike Pond (Miyagi Prefecture, N 38.439° E 140.919°), A5 from Furuichi Oike Pond (Tottori Prefecture, N 35.391° E 133.339°), A6 from Arigatani-ike Pond (Shizuoka Prefecture, N 34.691° E 138.126°), and B from Daizahoshi-ike Pond (Nagano Prefecture, N 36.706° E 138.145°) [7].

*Scenedesmus obliquus* algae was cultured in a flow-through system with COMBO [74], a defined freshwater culture medium for algae and zooplankton, and was used as the food source for *Daphnia* cultures. Before their use, algal cells were harvested, and their cell density was quantified under an optical microscope (Olympus, Tokyo, Japan). According to the previously measured carbon content for an algal cell of *S. obliquus* (2.09 × 10^− 8^ mg C cell^− 1^), the appropriate amounts of algae were estimated for achieving a designed carbon food level and then used in experiments.

Individuals in each genotype, taken from a single mother that originated from genotypes maintained for several years in our laboratory, were cultured in 900 ml bottles containing 600 ml of COMBO with 2.0 mg C L^− 1^ of *S. obliquus* in a controlled room (20 °C, photoperiod, Light(L): Dark(D) 14:10). The individuals were transferred to fresh medium every other day and fed daily. The individual abundance in the culture bottles was adjusted to less than 1 individual 20 ml^− 1^ in all the clones. Then, neonates born within 24 h (hr) were collected from the 3rd brood produced by the cultured individuals and used for the following experiments.

### Assay of digestive enzyme activity

In each clone, 20 neonates were randomly chosen and individually placed into 50-ml stoppered bottles containing the growth medium COMBO with *S. obliquus*. Half of the *Daphnia* were grown at a food concentration of 2.0 mg C L^− 1^, and the other half were grown at 0.2 mg C L^− 1^. The animals were fed daily and moved to new medium every two days. After a 4-day cultivation, on day 5, 10 individuals of each treatment were randomly collected. At least 5 individuals were used for the measurements of body length and body weight, and the remaining individuals were used for measuring digestive enzyme activity.

For measurements of body length and weight, 5-day-old animals were rinsed twice in distilled water and placed under a microscope where media was removed until the animals were properly positioned. Using an Olympus DP20 camera (Olympus, Tokyo, Japan) mounted on an Olympus SZH10 stereomicroscope (Olympus, Tokyo, Japan), images of *Daphnia* were captured at a magnification of 40×, and their body length from the base of the tail-spine to the top of the head was subsequently measured using ImageJ software (National Institutes of Health, Bethesda, USA). Then, they were individually transferred into a pre-weighed aluminium boat, dried for 24 h at 60 °C, cooled for 12 h in a vacuum desiccator at room temperature, and weighed using a Mettler-Toledo UMX2 microbalance to the nearest 0.1 μg (Mettler-Toledo, Tokyo, Japan).

For assaying the digestive enzyme activities, the animals were rinsed several times with distilled water, homogenized with a micropestle in 200 μl of ice-cold Tris/HCl buffer (0.05 mol L^− 1^, pH 8), and centrifuged for 10 min (min) at 14,000× gravitational acceleration (g) using a KUBOTA 1120 centrifuge (Kubota, Tokyo, Japan). The supernatant of the centrifuged samples was treated as an extract of a single animal for the analysis of beta-glucosidase (EC no. 3.2.1.21), lipase (3.1.1.3 and others), alkaline phosphatase (3.1.3.1), arginine amino-peptidase (3.4.11.6) and alanine amino-peptidase (3.2.11.2). Assays were run at room temperature with 5 μl of substrate, 15 μl of sample extract and 230 μl of citrate-phosphate buffer (0.1 mol L^− 1^, pH 5) [75] for beta-glucosidase or Tris/HCl buffer for alkaline phosphatase (0.05 mol L^− 1^, pH 8) and other enzymes (0.05 mol L^− 1^, pH 7), according to the protocol developed by Knotz et al. [76]. The substrates used in the enzyme assays were 4-methylumbelliferyl beta-d-glucoside (Sigma M3633) for beta-glucosidase, 4-methylumbelliferyl butyrate for lipase (Sigma 19,362), L-arginine-7-amido-4-methylcoumarin hydrochloride (Sigma A2027) for arginine amino-peptidase, and L-alanine-4-methyl-7-coumarinylamide-trifluoroacetate (Sigma A4302) for alanine amino-peptidase. Stock solutions of these substrates were prepared in ethylene glycol monomethyl ether (5 mmol L^− 1^). The concentration of these substrates in the assays was 100 μmol L^− 1^. Fluorescence was measured at 360 nm (excitation) and 450 nm (emission) every 5 min for 35 min with a Fluoroskan Ascent microplate fluorometer (Thermo Fisher Scientific, Tokyo, Japan). Blanks were run in parallel. For preparing standard curves, 0~10 μmol L^− 1^ of 4-beta-methylubelliferone (MUF) and 7-amino-4-methylcoumarin (AMC) were used [76].

The water-soluble protein content of the animals was measured using a bicinchoninic acid assay (BCA) [77]. Bovine serum albumin (BSA: 0, 50 mg L^− 1^, 100 mg L^− 1^) was used as the standard. For these measurements, 100 μL of supernatant from the centrifuged sample or BSA was mixed with 750 μL of the working reagent of the bicinchoninic acid kit (BCA1 SIGMA-ALDRICH) and incubated for 2 h at 55 °C. The assays were read at 562 nm using a UV-1600 spectrophotometer (Shimadzu, Tokyo, Japan). Digestive enzyme activity was calculated corresponding to the average water-soluble protein content of each clone in a specific unit (nmol hr.^− 1^ mg_protein_^− 1^).

### Growth experiment

A growth experiment was performed at the two food concentrations mentioned above. For each food concentration, 10 neonates of each clone were individually grown in 50-ml stoppered bottles containing the growth medium COMBO with algal food as in the experiment for measuring digestive enzyme activities. In this study, the experiment lasted until they had produced the sixth brood. To ensure that the food particles were homogeneous in the suspension, the bottles were secured to a grazing wheel that rotated at a speed of 1 revolution per minute (rpm). The growth medium and algal food were changed every two days, and *Daphnia* were fed daily.

When the growth medium was changed, images of the animals were captured as above and used for measuring the body length from the base of the tail-spine to the top of the head, the tail-spine length and the number of eggs in the brood pouch. In this study, maturation was defined as the time when the eggs first appeared in the brood pouch. The frequency of moulting casts (exuviae) and the size of newly released neonates were also counted and removed from the bottle.

The von Bertalanffy growth curve was applied for estimating the asymptotic body length *L*_*∞*_ and growth coefficient *k* as follows:

$$ {L}_t={L}_{\infty}\times \left\{1-\exp \left[-k\left(t-{t}_0\right)\right]\right\} $$

where *L*_*t*_ is the body length at age *t* (days), *t*_*0*_ is the hypothetical age at ‘0’ body length.

### Genetic analysis

To estimate the phylogenetic relationships and the genetic distances among *Daphnia* clones, we used genetic data acquired from our whole-genome sequencing as described below. Fifty to seventy individuals of each clone were used for DNA extraction conducted using a Maxwell^(R)^ 16 instrument and Maxwell^(R)^ 16 LEV Plant DNA Kit (Promega). Construction of the library and sample sequencing were performed at the Beijing Genomics Institute (BGI) JAPAN (Kobe, Japan). The libraries were constructed by a unique method of BGI JAPAN (low input method) from more than 500 ng of DNA per sample. The libraries were sequenced on an Illumina Hiseq X™ Ten platform (Illumina, San Diego, CA, USA) using a paired-end 150 bp (PE150) strategy to obtain approximately 8 Gb data/sample (approximately 40x coverage). The data were filtered using MapReduce acceleration-supported (SOAPunke) software [78] with the following options: -n 0.1, −l 10, −q 0.5, −i and -A 0.5. Reads of the individual FASTQ files were mapped to the reference genome data of *D. pulex* isolate PA42 [79] using burrows-wheeler alignment (BWA) [80]. Removal of potential polymerase chain reaction (PCR) duplicates and detection of polymorphisms in the data were conducted using sequence alignment/map(SAM) tools [81]. The proportion of different sites (uncorrected p-distance) was calculated using sequence data (135,933,993 base-pairs (bps)) with a > 20 quality score as the pairwise genetic distances among clones. To clarify the phylogenetic relationship among clones, we constructed an unrooted phylogenetic tree by the maximum likelihood (ML) method using SNP data with SNPhylo [82]. In this tree, we included several genotypes used in So et al. [7] other than JPN1 clones: *D. pulex* JPN2 (HO01) was collected from Lake Hataya Ohnuma (Yamagata Prefecture, N 38.245° E 140.204°), JPN3 (AWA) from Lake Awaji nariai-ji (Hyogo Prefecture, N 34.283° E 134.809°) and JPN4 (SUM) from Sumiyoshi ike Pond (Kagoshima Prefecture, N 31.772° E 130.592°), a genotype of *D. pulex* (LL05) was collected from a small lake in Manitoba, Canada (longitude/latitude unknown), and *D. pulicaria* (PUC01) was collected from Lake Biwa (Shiga Prefecture, N 35.176° E 136.979°).

### Statistical analysis

In this study, we measured a total of 17 phenotypic traits for each of the 5 clones (genotypes) that were grouped and categorized as enzyme activity (five traits), life history traits (seven traits) and morphological traits (five traits) (Table 1). A two-way analysis of variance (ANOVA) was used to examine the effects of genotype, food level, and their interaction (GxF) on each of the phenotypic traits. Significant effects were determined at *p* <  0.05, and α level was adjusted by a Bonferroni correction when multiple tests were performed. In these analyses, genotype and GxF were set as random factors, food level was set as a fixed factor, and variance components were estimated using the restricted maximum likelihood (REML) parameter. Before the analysis, a log-transformation was performed for beta-glucosidase, alkaline phosphatase, arginine amino-peptidase, maturation age, mean egg number of the first three clutches and body length at five days, and a two-step transformation algorithm [83] was performed for intermoult duration before maturation to stabilize the variance.

Broad-sense heritability (*H*^*2*^) for each trait was calculated as follows:

$$ {\mathrm{H}}^2={\mathrm{V}}_{\mathrm{g}}/{\mathrm{V}}_{\mathrm{T}}, $$

where V_g_ is the genetic variance and V_T_ is the total phenotypic variance [2]. The 95% confidence interval (CI) of heritability for each trait was estimated using the bootstrap method. The V_g_ was estimated by decomposing V_T_ using two-way ANOVA according to Holland et al. [84]. Then, the coefficient of genetic variation (CV) was calculated as follows:

$$ \mathrm{CV}={{\mathrm{V}}_{\mathrm{g}}}^{0.5}/\mu, $$

where *μ* is the mean value of given phenotypic traits.

To detect the relationships among traits, we first estimated the best linear unbiased predictors (BLUPs) of genotype values (sample number = 5 clones, Additional file 6: Table S5) for each trait based on the analysis above. Then, PCA was estimated using the BLUPs of heritable traits. Components with eigenvalues greater than 1.0 have been extracted to explain the variability of phenotypic traits. We used the BLUPs since these are more robust to unbalanced replication and less biased by environmental effects than the genotype mean values [85].

We examined the relationship between phenotypic dissimilarity and genetic distance among five genotypes. For this, we estimated the standardized Euclidean distance using the BLUPs of each trait. The distance was estimated for overall traits, traits in each category and single traits. Individual genetic distance was estimated using uncorrected p-distances by pairwise comparisons of the genome sequences between genotypes. Then, we examined the relationship between two matrices of genomic and phenotypic traits by a Mantel test.

These statistical tests were conducted with Statistical Product and Service Solutions (SPSS) statistics version 21.0 (International business machines corporation (IBM), Armonk, USA) and R version 3.3.3 [86].

## Additional files

Additional file 1: **Figure S1.** Phylogenetic relationships of five clones in JPN1 lineage and other clones. This is based on 5282 SNPs from whole genome sequence data. Using data with quality scores higher than 20, a phylogenetic tree of the clones by the maximum likelihood (ML) method were constructed using SNPhylo pipeline [82]. Numbers on branches indicate bootstrap values (> 50% are shown). In this analysis, we included genotypes used in So et al. [7] other than *D. pulex* JPN1 clones. These are *D. pulex* JPN2 (HO01), JPN3 (AWA) and JPN4 (SUM) and a genotype of *D. pulex* (LL05) collected from a small lake in Manitoba, Canada, and *D. pulicaria* (PUC01) collected from Lake Biwa. (PDF 199 kb)

Additional file 2: **Table S1.** Genetic distance measured by proportion of different sites (p-distance) among *Daphnia pulex* JPN1 clones (A1, A3, A5, A6 and B). (PDF 46 kb)

Additional file 3: **Table S2.** Distance of phenotypes in each category among *Daphnia pulex* JPN1 clones (A1, A3, A5, A6). The phenotypic distance was calculated using difference in BLUPs between clones. (PDF 51 kb)

Additional file 4: **Table S3.** Distance of single phenotypes among *Daphnia pulex* JPN1 clones (A1, A3, A5, A6 and B). The phenotypic distance was calculated using difference in BLUPs between clones. (PDF 68 kb)

Additional file 5: **Table S4.** Relationship between genetic distance and phenotypic distance between clones in each of the single traits. (PDF 55 kb)

Additional file 6: **Table S5.** BLUPs of each trait in *Daphnia pulex* JPN1 clones. (PDF 69 kb)

## Data Availability

Traits data used in this study are available on Dryad (doi: 10.5061/dryad.9p2j626). *Daphnia* genetic data are available from the authors upon request.

## Abbreviations

AMC: 7-amino-4-methylcoumarin
ANOVA: Analysis of variance
BCA: Bicinchoninic acid assay
BGI: The Beijing Genomics Institute
BLUPs: Best linear unbiased predictors
bps: Branch-point sequence
BSA: Bovine serum albumin
BWA: Burrows-wheeler alignment
CI: Confidence interval
COMBO: A defined freshwater culture medium for algae and zooplankton
CV: Coefficient of genetic variation
*D. pulex*: *Daphnia pulex*
E: Longitude
F: Food level
G: Genotype
g: Gravitational acceleration
GxF: Genotype by food level interaction
H^2^: Broad-sense heritability
hr.: Hour
IBM: International business machines corporation
*k*: Growth coefficient
L: D: Light: Dark
*L*_*∞*_: Asymptotic body length
L_t_: Body length at age t
min: Minute
ML: Maximum likelihood
mtDNA: Mitochondrial deoxyribonucleic acid
MUF: 4-beta-methylubelliferone
N: Latitude
PCA: Principal component analysis
PCR: Polymerase chain reaction
REML: Restricted maximum likelihood
rpm: Revolutions per minute
*S. obliquus*: *Scenedesmus obliquus*
SAMtools: Sequence alignment/map(SAM)tools
SNP: Single nucleotide polymorphism
SNPhylo: A pipeline to construct a phylogenetic tree from huge SNP data
SOAPunke: A MapReduce acceleration-supported software for integrated quality control and preprocessing of high-throughput sequencing data
SPSS: Statistical product and service solutions
SSR: Simple sequence repeat
t: Age (days)
t_0_: Hypothetical age at ‘0’ body length
V_g_: Genetic variation
V_T_: Total phenotypic variance
*μ*: Mean value of phenotypic traits
