# Supplementary material for: Evolution of asexual Daphnia pulex in Japan: variations and covariations of the digestive, morphological and life history traits
Source: BMC Evol Biol. 2019 Jun 13;19:122. doi: 10.1186/s12862-019-1453-9 (PMC6567566; doi:10.1186/s12862-019-1453-9)
Supplement: Supplementary file 1 — Figure S1. Phylogenetic relationships of five clones in JPN1 lineage and other clones. This is based on 5282 SNPs from whole genome sequence data. Using data with quality scores higher than 20, a phylogenetic tree of the clones by the maximum likelihood (ML) method were constructed using SNPhylo pipeline [82]. Numbers on branches indicate bootstrap values (> 50% are shown). In this analysis, we included genotypes used in So et al. [7] other than D. pulex JPN1 clones. These are D. pulex JPN2 (HO01), JPN3 (AWA) and JPN4 (SUM) and a genotype of D. pulex (LL05) collected from a small lake in Manitoba, Canada, and D. pulicaria (PUC01) collected from Lake Biwa. (PDF 199 kb) [file 12862_2019_1453_MOESM1_ESM.pdf]

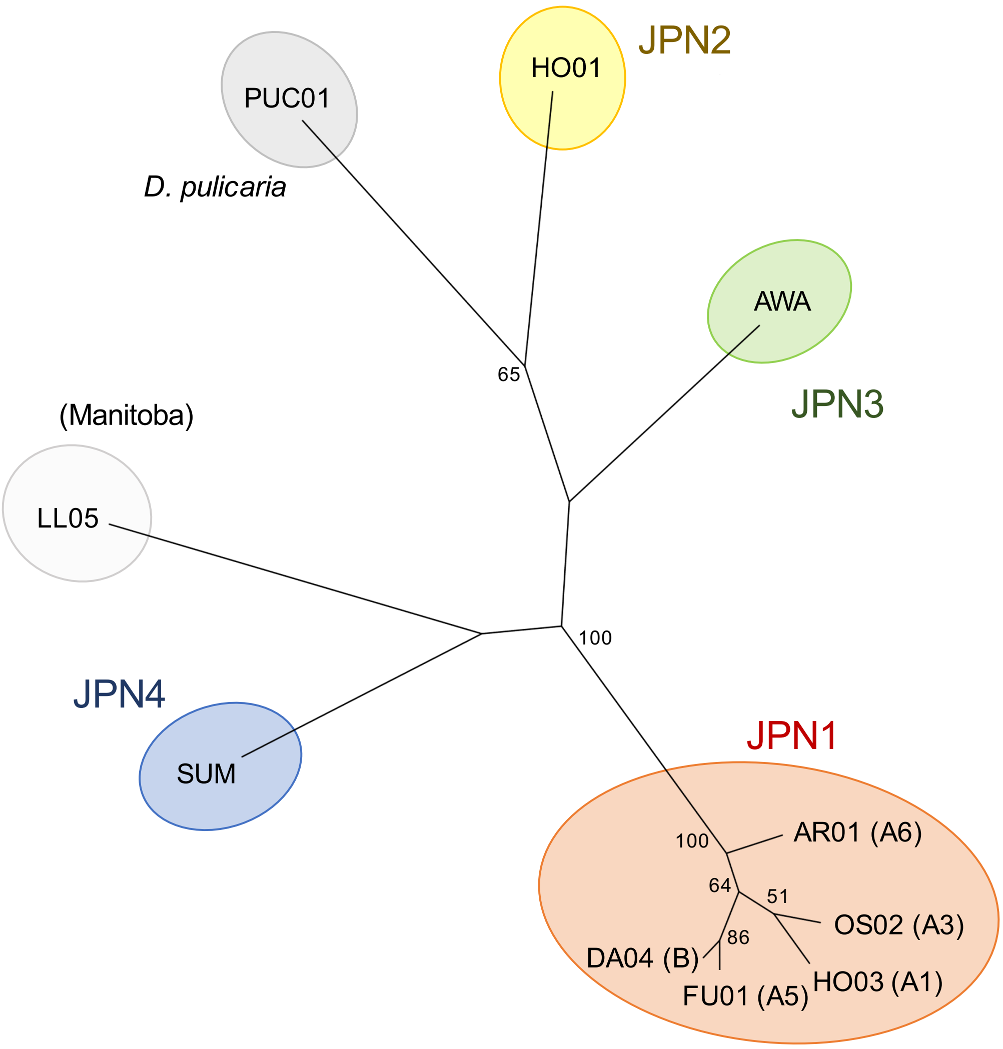

Figure S1. Phylogenetic relationships of five clones in JPN1 and other clones. This is based on 5282 SNPs from whole genome sequence data. Using data with quality scores higher than 20, a phylogenetic tree of the clones by the maximum likelihood (ML) method were constructed using SNPhylo pipeline [82]. Numbers on branches indicate bootstrap values (>50% are shown). In this analysis, we included genotypes used in So et al. [7] other than D. pulex JPN1 clones. These are *D. pulex* JPN2 (HO01), JPN3 (AWA) and JPN4 (SUM) and a genotype of *D. pulex* (LL05) collected from a small lake in Manitoba, Canada, and *D. pulicaria* (PUC01) collected from Lake Biwa.
