# Supplementary material for: Evolution of asexual Daphnia pulex in Japan: variations and covariations of the digestive, morphological and life history traits
Source: BMC Evol Biol. 2019 Jun 13;19:122. doi: 10.1186/s12862-019-1453-9 (PMC6567566; doi:10.1186/s12862-019-1453-9)
Supplement: Supplementary file 2 — Table S1. Genetic distance measured by proportion of different sites (p-distance) among Daphnia pulex JPN1 clones (A1, A3, A5, A6 and B). (PDF 46 kb) [file 12862_2019_1453_MOESM2_ESM.pdf]

Table S1. Genetic distance measured by proportion of different sites (p-distance) among *Daphnia pulex* JPN1 clones (A1, A3, A5, A6 and B).

Clone	A3	A5	A6	B
A1	1.75.E-03	1.66.E-03	1.85.E-03	1.72.E-03
A3		1.70.E-03	1.88.E-03	1.73.E-03
A5			1.92.E-03	1.41.E-03
A6				1.91.E-03
