# Supplementary material for: Evolution of asexual Daphnia pulex in Japan: variations and covariations of the digestive, morphological and life history traits
Source: BMC Evol Biol. 2019 Jun 13;19:122. doi: 10.1186/s12862-019-1453-9 (PMC6567566; doi:10.1186/s12862-019-1453-9)
Supplement: Supplementary file 3 — Table S2. Distance of phenotypes in each category among Daphnia pulex JPN1 clones (A1, A3, A5, A6). The phenotypic distance was calculated using difference in BLUPs between clones. (PDF 51 kb) [file 12862_2019_1453_MOESM3_ESM.pdf]

Table S2. Distances of phenotypes in each category among *Daphnia pulex* JPN1 clones (A1, A3, A5, A6 and B). The phenotypic distance was calculated using difference in BLUPs between clones.

		2.0 mg C L^{-1}				0.2 mg C L^{-1}				Combined			
Trait type	Clone	A3	A5	A6	B	A3	A5	A6	B	A3	A5	A6	B
Digestive	A1	3.25	3.24	3.54	4.03	3.27	2.46	1.93	4.28	3.07	2.19	2.34	4.22
	A3		2.13	0.97	1.52		2.56	2.35	3.35		2.20	2.10	3.43
	A5			2.44	3.20			1.77	2.11			2.16	2.52
	A6				1.08				3.10				3.24
Life history	A1	1.66	2.17	1.51	3.76	2.42	2.90	3.29	4.19	1.25	2.96	2.60	4.40
	A3		1.32	0.79	4.40		2.41	2.58	3.34		2.07	1.53	4.36
	A5			1.44	4.33			2.03	2.86			1.75	4.06
	A6				4.57				4.64				4.90
Morphology	A1	3.81	3.60	4.24	4.36	3.68	3.26	4.87	4.60	4.12	4.14	5.14	4.79
	A3		1.31	2.94	2.97		1.15	2.16	2.19		0.73	1.30	2.30
	A5			1.98	2.38			2.30	2.70			1.39	2.61
	A6				2.85				2.76				2.63
All	A1	5.28	5.31	5.73	7.03	5.63	5.17	6.31	7.66	5.28	5.54	6.21	7.75
	A3		2.72	3.43	5.47		3.72	4.12	5.22		3.15	3.11	6.11
	A5			3.46	5.89			3.56	4.48			3.11	5.45
	A6				5.49				6.22				6.44
