# Supplementary material for: Evolution of asexual Daphnia pulex in Japan: variations and covariations of the digestive, morphological and life history traits
Source: BMC Evol Biol. 2019 Jun 13;19:122. doi: 10.1186/s12862-019-1453-9 (PMC6567566; doi:10.1186/s12862-019-1453-9)
Supplement: Supplementary file 4 — Table S3. Distance of single phenotypes among Daphnia pulex JPN1 clones (A1, A3, A5, A6 and B). The phenotypic distance was calculated using difference in BLUPs between clones. (PDF 68 kb) [file 12862_2019_1453_MOESM4_ESM.pdf]

Table S3. Distance of single phenotypes among *Daphnia pulex* JPN1 clones (A1, A3, A5, A6 and B). The phenotypic distance was calculated using difference in BLUPs between clones.

		2.0 mg C L^{-1}				0.2 mg C L^{-1}				Mixture			
Trait	Clone	A3	A5	A6	B	A3	A5	A6	B	A3	A5	A6	B
Beta-glucosidase	A1	144.59	102.49	128.61	124.11	123.43	15.31	10.53	28.29	73.98	20.15	28.19	21.52
	A3		42.10	15.98	20.48		138.74	133.96	151.72		53.83	45.79	52.46
	A5			26.13	21.62			4.78	12.98			8.04	1.37
	A6				4.51				17.76				6.67
Lipase	A1	-	-	-	-	175.10	180.60	274.78	59.92	101.77	92.10	113.83	2.67
	A3		-	-	-		5.49	99.67	115.18		9.67	12.06	104.44
	A5			-	-			94.18	120.68			21.73	94.77
	A6				-				214.85				116.50
Alkaline phophatase	A1	16.93	55.86	22.51	65.96	106.72	273.04	2.34	716.08	26.35	35.13	7.30	163.17
	A3		72.79	5.58	49.03		166.32	109.06	609.36	0.00	8.78	19.05	136.82
	A5			78.37	121.82			275.38	443.04	8.78	0.00	27.84	128.04
	A6				43.45				718.42	19.05	27.84	0.00	155.88
Arginine amino-peptidase	A1	23.98	37.63	28.14	38.11	43.06	112.85	30.18	149.72	17.00	37.82	0.22	47.15
	A3		13.65	4.16	14.13		69.79	73.24	106.65		20.81	17.22	30.15
	A5			9.48	0.48			143.03	36.87			38.03	9.33
	A6				9.97				179.90				47.36
Alanine amino-peptidase	A1	16.96	12.86	47.15	47.06	43.16	36.63	58.65	82.46	32.19	26.75	58.39	70.83
	A3		4.11	30.19	30.10		6.53	15.49	39.30		5.44	26.19	38.63
	A5			34.30	34.20			22.02	45.83			31.64	44.08
	A6				0.09				23.81				12.44
Maturation age	A1	0.29	0.1	0.03	1.39	0.12	0.28	0.2	1.14	0.23	0.26	0.14	1.47
	A3		0.19	0.32	1.1		0.17	0.32	1.02		0.03	0.37	1.24
	A5			0.13	1.29			0.48	0.86			0.4	1.2
	A6				1.42				1.34				1.61
Maturation instar number	A1	0.07	0.05	0.05	0.39	0.24	0.25	0.33	0.65	0.09	0.18	0.15	0.65
	A3		0.03	0.03	0.31		0.49	0.09	0.89		0.27	0.06	0.74
	A5			0.00	0.34			0.58	0.4			0.33	0.46
	A6				0.34				0.98				0.79
Maturation body length	A1	0.05	0.05	0.07	0.06	0.04	0.07	0.12	0.01	0.05	0.08	0.12	0.03
	A3		0.0007	0.02	0.11		0.03	0.08	0.03		0.02	0.06	0.08
	A5			0.02	0.11			0.05	0.06			0.04	0.1
	A6				0.13				0.11				0.14
Intermoult duration before maturation	A1	0.00	0.00	0.00	0.00	0.09	0.07	0.04	0.02	0.02	0.01	0.01	0.01
	A3		0.00	0.00	0.00		0.16	0.05	0.07		0.03	0.01	0.01
	A5			0.00	0.00			0.11	0.09			0.02	0.02
	A6				0.00				0.01				0.001
Intermoult duration after maturation	A1	0.04	0.01	0.04	0.001	0.1	0.14	0.12	0.1	-	-	-	-
	A3		0.05	0.08	0.04		0.04	0.02	0.0006		-	-	-
	A5			0.03	0.01			0.01	0.04			-	-
	A6				0.04				0.02				-
k	A1	0.0022	0.0072	0.0009	0.0177	0.0161	0.0384	0.0329	0.0376	0.0061	0.0223	0.0149	0.0276
	A3		0.0094	0.0012	0.0198		0.0224	0.0168	0.0215		0.0163	0.0089	0.0216
	A5			0.0081	0.0105			0.0055	0.0008			0.0074	0.0053
	A6				0.0186				0.0047				0.0127
Mean egg number of the first three clutches	A1	1.72	2.46	1.07	1.25	0.85	0.24	0.16	0.96	0.11	0.37	0.14	0.43
	A3		0.74	0.66	2.98		0.61	0.69	0.11		0.26	0.03	0.54
	A5			1.4	3.72			0.08	0.72			0.23	0.8
	A6				2.32				0.8				0.57
Mean neonate size of the first three clutches	A1	0.0063	0.0044	0.0008	0.0033	0.079	0.1079	0.1317	0.0821	0.005	0.0058	0.0058	0.0044
	A3		0.0019	0.0071	0.003		0.0289	0.0528	0.0031		0.0008	0.0008	0.0006
	A5			0.0052	0.001			0.0238	0.0258			2E-06	0.0013
	A6				0.0041				0.0496				0.0014
Body length at five days	A1	0.27	0.27	0.32	0.31	0.20	0.12	0.27	0.21	0.25	0.21	0.32	0.28
	A3		0.01	0.06	0.05		0.04	0.04	0.03		0.04	0.07	0.03
	A5			0.05	0.04			0.16	0.10			0.11	0.07
	A6				0.01				0.06				0.04
Body weight at five days	A1	0.003	0.01	0.01	0.01	0.003	0.003	0.01	0.01	0.003	0.01	0.01	0.01
	A3		0.005	0.007	0.009		0.0004	0.005	0.003		0.002	0.006	0.006
	A5			0.002	0.005			0.005	0.004			0.004	0.004
	A6				0.002				0.001				0.0003
Relative tail spine length of the first adult instar	A1	0.08	0.07	0.09	0.09	0.07	0.06	0.05	0.06	0.08	0.06	0.07	0.08
	A3		0.02	0.01	0.01		0.02	0.02	0.01		0.02	0.004	0.0008
	A5			0.03	0.02			0.002	0.01			0.01	0.01
	A6				0.003				0.01				0.003
$L\infty$	A1	0.18	0.2	0.27	0.17	0.01	0.004	0.01	0.09	0.06	0.07	0.1	0.12
	A3		0.02	0.08	0.35		0.01	0.02	0.07		0.02	0.05	0.18
	A5			0.07	0.37			0.01	0.09			0.03	0.2
	A6				0.44				0.1				0.23
