# Supplementary material for: Evolution of asexual Daphnia pulex in Japan: variations and covariations of the digestive, morphological and life history traits
Source: BMC Evol Biol. 2019 Jun 13;19:122. doi: 10.1186/s12862-019-1453-9 (PMC6567566; doi:10.1186/s12862-019-1453-9)
Supplement: Supplementary file 5 — Table S4. Relationship between genetic distance and phenotypic distance between clones in each of the single traits. (PDF 55 kb) [file 12862_2019_1453_MOESM5_ESM.pdf]

Table S4. Relationship between genetic distance and phenotypic distance between clones in each of the single traits.

Trait		2.0 mg C L $^{-1}$	0.2 mg C L $^{-1}$	Combined
Digestive				
Beta-glucosidase	*r*	-0.06	0.05	0.12
	p	0.52	0.32	0.30
Lipase	*r*	-	0.23	-0.15
	p	-	0.28	0.64
Alkaline phosphatase	*r*	-0.67	-0.15	-0.28
	p	0.96	0.59	0.63
Arginine amino-peptidase	*r*	0.02	0.41	0.26
	p	0.37	0.07	0.15
Alanine amino-peptidase	*r*	-0.05	-0.27	-0.18
	p	0.55	0.73	0.66
Life history				
Maturation age	*r*	-0.32	-0.07	-0.18
	p	0.64	0.49	0.62
Maturation instar number	*r*	-0.38	0.16	-0.08
	p	0.80	0.31	0.52
Maturation body length	*r*	-0.28	0.33	0.03
	p	0.76	0.18	0.51
Intermoult duration before maturation	*r*	-0.19	-0.31	-0.37
	p	0.70	0.75	0.79
Intermoult duration after maturation	*r*	0.56	-0.14	-
	p	0.15	0.59	-
k	*r*	-0.14	0.01	-0.04
	p	0.58	0.36	0.54
Mean egg number of the first three clutches	*r*	-0.63	-0.22	-0.63
	p	0.98	0.74	0.92
Morphology				
Mean neonate size of the first three clutches	*r*	0.49	0.14	-0.11
	p	0.05	0.32	0.56
Body length at five days	*r*	-0.02	0.15	0.05
	p	0.44	0.35	0.36
Body weight at five days	*r*	-0.11	0.22	-0.03
	p	0.62	0.28	0.57
Relative tail spine length of the first adult in	*r*	-0.06	-0.05	-0.09
	p	0.52	0.49	0.54
L_{∞}	*r*	-0.24	-0.29	-0.27
	p	0.74	0.61	0.73
