# Supplementary material for: Evolution of asexual Daphnia pulex in Japan: variations and covariations of the digestive, morphological and life history traits
Source: BMC Evol Biol. 2019 Jun 13;19:122. doi: 10.1186/s12862-019-1453-9 (PMC6567566; doi:10.1186/s12862-019-1453-9)
Supplement: Supplementary file 6 — Table S5. BLUPs of each trait in Daphnia pulex JPN1 clones. (PDF 69 kb) [file 12862_2019_1453_MOESM6_ESM.pdf]

Table S5. BLUPs of each trait in *Daphnia pulex* JPN 1 clones.

Food concentration	Clone	Beta-glucosidase	Lipase	Alkaline Phosphatase	Arginine amino-peptidase	Alanine amino-peptidase	Maturation age	Maturation instar number	Maturation body length	Intermoult duration before maturation	Intermoult duration after maturation	*k*	Mean egg number of the first three clutches	Mean neonate size of the first three clutches	Body length at five days	Body weight at five days	Relative tail spine length of the firstt adult instar	$L\infty$
	A1	99.96	0.00	9.91	25.57	24.81	0.35	0.11	-0.02	1.26E-16	0.004	-0.00435	-0.8	-0.00263	-0.2345	-0.00674	-0.066	-0.1
	A3	-44.63	0.00	-7.02	1.59	7.84	0.06	0.04	0.03	-1.58E-16	0.04	-0.00652	0.92	0.00365	0.031306	-0.00353	0.017	0.09
2.0 mg C [illegible]	A5	-2.53	0.00	65.77	-12.06	11.95	0.25	0.06	0.03	6.84E-17	-0.008	0.00285	1.66	0.001719	0.03734	0.001138	0.001	0.1
	A6	-28.65	0.00	-12.60	-2.57	-22.35	0.38	0.06	0.05	6.84E-17	-0.038	-0.00528	0.27	-0.00343	0.087577	0.003487	0.026	0.17
	B	-24.15	0.00	-56.05	-12.54	-22.25	-1.04	-0.28	-0.08	-1.05E-16	0.002	0.013304	-2.05	0.000696	0.078273	0.005653	0.023	-0.27
	A1	13.86	138.08	218.70	55.09	44.18	0.27	0.06	-0.05	0.02	-0.09	-0.02499	0.44	-0.08016	-0.15982	-0.00384	-0.049	0.02
	A3	-109.57	-37.02	111.98	12.03	1.02	0.15	0.31	-0.01	-0.07	0.01	-0.00893	-0.41	-0.00117	0.038599	-0.00092	0.022	0.01
0.2 mg C [illegible]	A5	29.17	-42.52	-54.34	-57.76	7.55	-0.01	-0.18	0.02	0.08	0.04	0.013427	0.2	0.027777	-0.04346	-0.00127	0.007	0.02
	A6	24.39	-136.70	221.04	85.27	-14.47	0.47	0.39	0.07	-0.02	0.03	0.007885	0.28	0.051587	0.112982	0.003746	0.005	0.03
	B	42.15	78.16	-497.38	-94.63	-38.28	-0.87	-0.58	-0.04	-0.01	0.01	0.012606	-0.52	0.001967	0.051698	0.002288	0.015	-0.07
	A1	28.77	61.01	46.39	20.35	37.63	0.36	0.12	-0.04	0.004	0	-0.01419	-0.04	-0.0042	-0.20913	-0.0054	-0.057	-0.02
	A3	-45.21	-40.77	20.04	3.35	5.44	0.14	0.21	0.01	-0.013	0	-0.00813	0.07	0.000804	0.038016	-0.00221	0.019	0.03
Combined	A5	8.61	-31.09	11.26	-17.47	10.88	0.1	-0.07	0.03	0.014	0	0.008132	0.33	0.001582	-0.00389	-8.60E-05	0.004	0.05
	A6	0.58	-52.82	39.09	20.57	-20.75	0.5	0.26	0.07	-0.002	0	0.00072	0.1	0.001584	0.106485	0.003703	0.015	0.08
	B	7.25	63.67	-116.78	-26.80	-33.20	-1.1	-0.53	-0.07	-0.003	0	0.013465	-0.47	0.000234	0.068525	0.003997	0.019	-0.15
